# Is the use of RAS inhibitors safe in the current era of COVID-19 pandemic?

**DOI:** 10.1186/s40885-020-00144-0

**Published:** 2020-05-07

**Authors:** Sungha Park, Hae Young Lee, Eun Joo Cho, Ki Chul Sung, Juhan Kim, Dae-Hee Kim, Sang-Hyun Ihm, Kwang-il Kim, Il-Suk Sohn, Wook-Jin Chung, Hyeon Chang Kim, Sung Kee Ryu, Wook Bum Pyun, Jinho Shin, on behalf of the Korean Society of Hypertension

**Affiliations:** 1 Division of Cardiology, Severance Cardiovascular Hospital and Severance Cardiovascular Hospital and Integrated Research Center for Cerebrovascular and Cardiovascular diseases, Yonsei University College of Medicine, Seoul, South Korea; 2 Division of Cardiology, Department of Internal Medicine, Seoul National University Hospital, Seoul, South Korea; 3 Division of Cardiology, Department of Internal Medicine, Yeouido St. Mary’s Hospital., College of Medicine, The Catholic University of Korea, Seoul, South Korea; 4 Division of Cardiology, Department of Internal Medicine, Kangbuk Samsung Hospital, Seoul, South Korea; 5 Division of Cardiology, Department of Internal Medicine, Chonnam National University Hospital, Gwangju, South Korea; 6 Department of Cardiology, Asan Medical Center, College of Medicine, Ulsan University, Seoul, South Korea; 7 Division of Cardiology, Department of Internal Medicine, Bucheon St. Mary’s Hospital., College of Medicine, The Catholic University of Korea, Seoul, South Korea; 8 Division of Geriatrics, Department of Internal Medicine, Seoul National University Bundang Hospital, Seongnam, South Korea; 9 Division of Cardiology, Department of Internal Medicine, Kyung Hee University at Gangdong, Seoul, South Korea; 10 Division of Cardiology, Department of Internal Medicine, Gil Hospital, Gachon University, Incheon, South Korea; 11 Department of Preventive Medicine, Yonsei University College of Medicine, Seoul, South Korea; 12 Division of Cardiology, Department of Internal Medicine, Nowon Eulji Medical Center, Eulji University, Seoul, South Korea; 13 Division of Cardiology, Department of Internal Medicine, Ewha Womans University Seoul Hospital, Seoul, South Korea; 14 Division of Cardiology, Department of Internal Medicine, Hanyang University Medical Center, 222 Wangsimni-ro Sungdong-gu, Seoul, South Korea

**Keywords:** Hypertension, Infection, SARS, COVID-19, 2019 novel coronavirus, SARS-CoV-2, Sepsis, Pandemic, Antihypertensive drugs, ACE inhibitor, Angiotensin receptor blocker, ACE2

## Abstract

Antihypertensive drugs are one of the most widely used pharmacologic agent in the world and it is predominantly used in the elderly subjects. Pneumonia is the most common cause of death in the extremely old subject. During infection and its complication such as sepsis, hypotension could be exacerbated by antihypertensive drugs because homeostasis mechanisms such as sodium balance, renin angiotensin aldosterone system and/or sympathetic nervous system can be mitigated by antihypertensive drug therapy. Severe Acute Respiratory Syndrome-Coronavirus-1 and 2 viral surface protein is known to attach angiotensin converting enzyme 2 (ACE2) on the cell membrane to facilitate viral entry into the cytoplasm. Despite the theoretical concerns of increased ACE2 expression by Renin-Angiotensin-Aldosterone system (RAS) blockade, there is no evidence that RAS inhibitors are harmful during COVID-19 infection and have in fact been shown to be beneficial in animal studies. Therefore, it is recommended to maintain RAS blockade during the current corona virus pandemic.

## Background

In the era of extremely aging society, hypertension is a highly prevalent chronic condition in the elderly that will overlap with many acute conditions such as infection, surgery, trauma or intoxication. Infection is an unavoidable clinical problem in the elderly patient taking antihypertensive medications. Pneumonia and influenza could be fatal in the elderly patients with cardiovascular complications, which are the result of long-term hypertension and other risk factors. In fact, vaccination against pneumonia and influenza is highly recommended in elderly with or without cardiovascular complications. Recent pandemic outbreak of Severe Acute Respiratory Syndrome corona virus 2 (SARS-CoV-2), so called, coronavirus disease 2019 (COVID-19) are posing a similar challenge in which the majority of fatalities are being observed in the elderly with hypertension, diabetes and/or their complications.

Antihypertensive drug therapy may have an impact on the clinical practice according to the wide variety of patient conditions. It will vary from mild conditions such as poor oral intake, volume depletion, orthostatic hypotension to severe conditions such as sepsis, septic shock, myocardial damage, overt hear failure, acute kidney injury, and/or multiple organ failure.

The changes in antihypertensive drug therapy should be based on weighing the risk benefit ratio. But there are few specific recommendations to guide the use of antihypertensive drugs in terms of vulnerability to specific infection, septic shock, and/or organ failure.

## General aspects of infection and hypertension management

There are some general aspects in relation to hypertension management or antihypertensive drug use when some infection is impending or ongoing. First of all, because hypertension is basically dependent on the sodium balance, when sodium or food intake is stopped, there could be an increased risk for unexpected blood pressure decrease and hypotension. Negative sodium balance can be exacerbated by febrile condition and the use of potent diuretics. In this situation, increase in renin-angiotensin-aldosterone system (RAS) activation and sympathetic nervous system acts as a compensatory mechanism to mitigate hypotension. As such, the patient taking potent or long acting RAS blockade or beta blocker might be vulnerable to hemodynamic instability compared to calcium antagonist. For this issue, diuretics does not have blocking effect of compensatory system but it could exacerbate underlying sodium depleting situation. These theoretical speculations might be one of possible explanation for the reason why calcium antagonist have superior profile in term of inter-individual visit to visit blood pressure variabilities. But, despite of all these speculations, there is few studies to answer this questions and clinical practice when a patient has asymptomatic hypotension or orthostatic hypotension is solely dependent on the physician’s discretions. Because RAS blockade or beta blocker is recommended for the patient with cardiovascular complications, stopping these drugs may be harmful. In a recent retrospective population based cohort study, Hsu W, et al., reported that established RAS blockade therapy before sepsis will be protective for the sepsis related mortality [1]. These results seems to be in line with the recent study showing depressed sympathetic modulation can be a signal for sepsis in patient with infection [2]. Some mediators to suppress sympathetic nervous system may be required for sustained hypotension so that those mediators may not be effective if additional blocking is not possible due to already established sympathetic blockade. Similarly, in retrospective studies for heart failure patients, withdrawal of RAS blockade or beta blocker were associated with poor prognosis [3, 4]. As such, there is no evidence to stop using these drugs in infection patients unless they are associated with hemodynamic instability. However, in patients with volume depletion and symptomatic hypotension, it would be prudent to stop antihypertensive medications and diuretics until blood pressure is normalized and the volume status is recovered. In patients who are hemodynamically stable, temporarily stopping RAS blockade or beta blocker seems to be possible only when the patient has no hypertension related complications such as heart failure, coronary artery disease, and other vascular complications. Even in these subjects, one should consider the adverse events resulting from rebound increase in blood pressure. Moreover, in the study by Ohkubo et al., including 6105 patients with a history of stroke and/or transient ischemic attack (mean age, 64 years), it was reported that perindopril decreased pneumonia risk in Asian but not in non-Asian subjects [5].

## Septic shock and antihypertensive drugs

Even with those retrospective studies showing that stopping RAS blockade or beta blocker is associated with poor prognosis, it is not usual to use vasoconstrictor or inotropic without temporary withdrawal of RAS blockade or beta blocker in a real world clinical practice for the patient with acute hypotension, acute kidney injury, or hyperkalemia related to infection [6]. Besides the toxin supplied by infectious organisms, catecholamine or stress induced cardiomyopathy, tachycardia induced cardiomyopathy, and direct invasion of infectious agents could precipitate hypotension during infection. Acute hemodynamic instability or hypotension is a serious condition during acute infection which requires stopping or adjustment of antihypertensive drug dosage according to the patient’s hemodynamic status.

## Pandemic viral diseases

Yearly outbreak of the seasonal flu is a huge burden on the healthcare system and survival in extremely older patients or in patients with high risk profiles such as older age, diabetes, chronic diseases of the heart, kidney, lung, and liver [7]. Fortunately, there are key available strategies to cope with it, which are specific to the infectious agent such as anti-viral therapy, vaccination, and other preventive approaches. Hypertension is prevalent in high risk flu patients because they are usually older and with cardiovascular complications. But antihypertensive agents could be adjusted according to the general and hemodynamic conditions only after infection begins and it will be only temporary even when the drugs need adjustments. There is no issue about adjustment of antihypertensive drug use to protect from infection preemptively because there is no direct involvement of antihypertensive drugs in the infectious process itself.

In contrast to pandemic flu, it is highlighted that angiotensin converting enzyme 2 (ACE2) of the lung type 2 alveolar epithelial cell acts as a non-catalytic receptor for SARS-CoV-1 and presumably for SARS-CoV-2 to infect the cell [8]. ACE2 is reported to be predominantly localized on endothelial cells. Its mRNA is reported to be most highly expressed in testis, kidney, and, heart and also expressed in brain, intestine, and lungs [9]. Thus, there have been growing concerns about hypertension being a risk factor for severe COVID-19 and the possibility of RAS inhibitors being an aggravating factor of severe COVID-19 due to the theoretical increase in the expression of systemic ACE2 with its use. The pandemic of SARS-CoV-2 which began in Wuhan, China from December 2019, has spread rapidly around the world. Severe lung damage and high mortality rates have been reported, particularly in the elderly and those with comorbidities. In particular, the high prevalence of hypertension and diabetes suggests that they maybe risk factors for severe COVID-19. The prevalence of hypertension is apparently high in patients with severe COVID-19, however, considering the age of those patients, the actual prevalence of hypertension does not seem to be higher. Zhou et al. analyzed the clinical characteristics of 191 patients diagnosed in Wuhan. Among them, the average age of deaths (*n* = 54) was 69 years, and the prevalence of hypertension was 48%, which is quite comparable to the prevalence of hypertension in the representative population of those ages [10]. Until now, none of the papers published through multivariate analysis indicated that hypertension was an independent risk factor for severe COVID-19. In the study by Guan et al., reporting the clinical features of 1099 COVID-19 patients diagnosed in Wuhan, the prevalence of hypertension was higher in severe patients (*N* = 173, mean age 52 years) than in mild patients (mean age 45 years)(23.7% vs. 13.4%), but it was not particularly higher when considering the prevalence of hypertension in that age group [11]. Therefore, there is no scientific evidence to suggest that hypertension itself is an independent risk factor for severe infection or mortality by COVID-19. The higher prevalence of hypertension in patients with COVID-19 is more likely due to the fact that the age of severe patients was significantly older and because major complications of hypertension such as chronic heart failure, cerebral infarction, and chronic renal failure make the patient much more vulnerable to progression to severe infection or death.

**Fig. 1:**
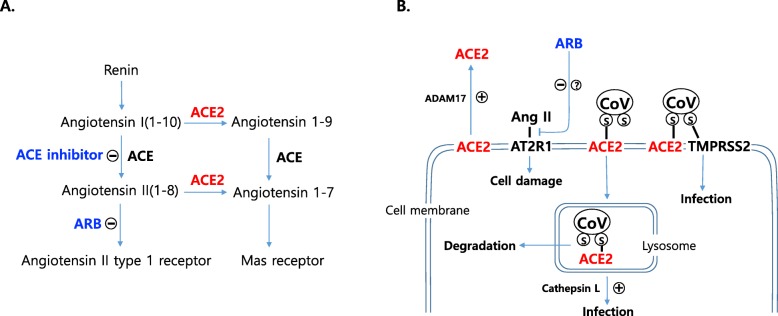
Angiotensin Converting Enzyme 2 in Classical Renin Angiotensin System and SARS-CoV-1. Panel **a**. In classical renin angiotensin system, ACE2 convert angiotensin I and II to Angiotensin 1–9 and 1–7, respectively. Panel **b**. Complex interaction among SARS-CoV-1, ACE2, TMPRSS2, and ADAM17 in lung epithelial cells. In SARS-CoV-1 infection and presumably in SARS-CoV-2 infection, binding of spike protein to ACE2 together with lysosome degradation resulting in ACE2 down regulation, ADAM17 mediated ACE2 shedding from cell membrane resulting in promotion of viral entry, facilitation of ACE2 mediated viral entry by TMPRSS2 make a complex expression or interplay between ACE2 and the virus. ACE, angiotensin converting enzyme; ADAM17, a disintegrin and metallopeptidase domain 17; ARB; angiotensin receptor blocker; S, spike protein; SARS-CoV, severe acute respiratory syndrome corona virus; TMPRSS2, The type II transmembrane serine proteases

In 2004, since the SARS-CoV-1 outbreak in Asia, the coronavirus has been shown to use ACE2 proteins in the epithelial cell membrane in the lung alveoli as a receptor. The viral spike(S) glycoprotein attaches to the ACE2 proteins which have been shown to facilitate viral entry and in theory, make the patient vulnerable to lower respiratory tract infection or viral pneumonia [12, 13]. Since the COVID-19 outbreak, role of ACE2 in the pathophysiology of COVID-19 has drawn a lot of interest in the social media world as well as scientific groups due to the growing concerns that increased expression of ACE2 may increase the risk for severe COVID-19. In a recent report, the main receptor of the spike (S) glycoprotein of SARS-CoV-2 is ACE2 and its affinity has been reported to be higher than that of SARS-CoV 1 [14]. So the key point of concern is that RAS inhibitor (ACE inhibitor, angiotensin receptor blocker), a drug commonly used in hypertensive patients, can theoretically raise ACE2 expression by a classical feedback system in the biochemical pathway when an enzyme or receptor was blocked (Fig. 1, a). When ACE2 is activated, Angiotensin 1–7 is increased instead of Angiotensin II. Angiotensin 1–7 is known to have an effect of protecting cardiovascular diseases by binding to the receptor Mas receptor to promote vasodilation, antioxidant and anti-inflammatory effects [15]. On the other hand, when it comes to COVID-19, the possibility exists that ACEI or ARB can be an exacerbation factor by increasing the expression of ACE2. As such, there have been increased incidence where patients or physicians change the antihypertensive medications to other drugs or discontinue the drugs due to concerns about the concerns, whether real or not, for increased risk of severe COVID-19. However, the Korean society of Hypertension would like to emphasize that currently, there is no scientific basis for taking such measures. First, there is no epidemiologic evidence for increased severe COVID-19 or mortality in patients who were taking RAS inhibitors. Second, it is still unclear whether or not RAS inhibition results in increased expression of ACE2 in systemic level and/or tissue including epithelial cells (Fig. 1). Even though there are reports that the concentration of ACE2 in the blood rises after administration of the first RAS blockade, there are conflicting reports that also suggest otherwise [16, 17]. Third, since ACE2 is a receptor for SARS-CoV-1 and 2, if RAS blockade does indeed exacerbates corona virus infection, obvious increase in mortality and severe infection in patients receiving RAS inhibitor should have been reported during the SARS-CoV-1 epidemic. As we all know, this was obviously not the case. On the contrary, it has been reported that ACE2 protects against severe acute lung injury. Study by Deshotels et al. have shown that Angiotensin II, which is highly activated in severe infection, results in increased ACE2 internalization and ACE2 degradation [18]. This has importance implications as the interaction between ACE2 and SARS-CoV-1 is very complex and the net expression of ACE2 cannot be predictable in a real patient (Fig 1, b) [19]. Moreover, in vivo studies have shown that decreased ACE2 during SARS-CoV-1 infection exacerbated acute lung injury. Imai et al. conducted experiments by inducing acute lung injury through gastric acid aspiration in mice. In ACE knockout mice, acute lung injury was significantly inhibited, whereas in ACE2 knockout mice, acute lung injury worsened compared to wild type. On the other hand, acute lung injury was significantly improved by recombinant ACE2 administration, demonstrating the protective effect of ACE2 in acute lung injury [20]. In addition, Kuba et al. conducted experiments with the mice SARS-CoV-1 model. The spike protein of SARS-CoV-1 attaches to ACE2 and downregulates ACE2 and activates Angiotensin II to exacerbate acute lung injury. In contrast, RAS blockade with losartan 15 mg/kg had a protective effect against acute lung injury caused by SARS-CoV-1 infection (Fig 1, b). Given that COVID-19 has similar pathophysiological mechanisms to SARS-CoV-1 infection, increase in ACE2 or RAS blockade might even be protective against severe infection or death.

In summary, 1) the prevalence of hypertension in patients with severe COVID-19 is high. However, it is more likely due to the fact that severe patients tend to be older and commonly have complications of hypertension, such as heart failure, coronary artery disease, stroke and CKD. Currently, there is no scientific evidence that hypertension is an independent risk factor for severe infection or death until proven otherwise. 2) Regarding the concerns for increased risk of severe infection or death due to the theoretical concerns that taking RAS inhibitors may increase lung ACE2, there is no clinical evidence to suggest that patients taking RAS inhibitors may have adverse prognosis during COVID-19. In fact, it is possible that RAS blockade has protective effect against severe COVID-19 based on previous reports of their protective effect in animal models of SARS-CoV-1 infection. 3) If RAS inhibitors administered due to high blood pressure or other cardiovascular disease were stopped abruptly, there is risk for excessive increase in blood pressure that may precipitate cardiovascular complications. However, we do acknowledge that the above-mentioned issues are valid concerns that deserve more research as quickly as possible. But without any scientific evidence to suggest otherwise, we the Korean Society of Hypertension endorse the recent recommendation by the European Society of Cardiology and recommend 1) In hemodynamically stable patients who were taking RAS inhibitors to be maintained on RAS blockade 2) In hemodynamically stable, treatment naïve patients who have compelling indication for using RAS inhibitors to be administered with RAS inhibitors according to the current guidelines 3) In patients without COVID-19 to be prescribed with RAS inhibitors if there are clinical indications to do so. 4) In hemodynamically unstable patients with COVID-19, prudent reduction or withdrawal of antihypertensive medications can be considered with consideration for the comorbidities and the indications for using the drug(s) before the infection.

## Conclusions

In conclusion, the use or temporary adjustment of antihypertension drugs during infectious disease could be individualized according to volume status, hemodynamic stability, comorbid cardiovascular disease profiles, and antihypertensive drug class. Also, the use of RAS blockade SARS-CoV-2 pandemic before infection should be maintained based on the current scientific knowledge. More researches are needed for the use of antihypertensive drugs before and during severe infection or epidemics.

## Data Availability

Not applicable.
